# Polyhydramnios and lithium disposition in pregnancy: clinical insights from a retrospective observational cohort study

**DOI:** 10.1192/j.eurpsy.2026.10187

**Published:** 2026-07-31

**Authors:** M. L. Imaz, L. Cabral, M. Torra, L. Klaus, E. Poch, E. Vieta, R. Martin-Santos, O. Gomez

**Affiliations:** 1 Unit of Perinatal Mental Health. Department of Psychiatry and Psychology. Institute of Neuroscience (ICN), Hospital Clinic; 2 Institut d’Investigacions Biomèdiques August Pi i Sunyer (IDIBAPS); 3 BCNatal Fetal Medicine Research Center (Hospital Clínic and Hospital Sant Joan de Déu); 4 Pharmacology and Toxicology Laboratory. Biochemistry and Molecular Genetic Service. Biomedical Diagnostic Center (CBD), Hospital Clinic; 5 Statistics and Operational Research, Universitat Politècnica de Barcelona; 6 Integrative Pharmacology and Systems Research Group, Hospital del Mar Research Institute; 7 Nephrology and Renal Transplant Service. Institute of Nephrology and Urology (ICNU), Hospital Clinic; 8 Medicine, Universitat de Barcelona; 9 Psychiatry and Psychology, Hospital Clinic; 10 Centro de Investigación Biomédica en Red en Salud Mental (CIBERSAM); 11 Maternofetal Service. Institute of Gynecology, Obstetric and Neonatology (ICGON), Hospital Clinic, Barcelona, Spain

## Abstract

**Introduction:**

Lithium is a first-line mood stabilizer utilized in the management of perinatal bipolar disorder. Lithium is primarily excreted renally. Pregnancy induces significant physiological adaptations, including expanded plasma volume and altered renal function, which can impact lithium disposition. Polyhydramnios, a pathological increase in amniotic fluid volume, may further modulate maternal fluid dynamics and renal perfusion, thereby influencing lithium pharmacokinetics.

**Objectives:**

To characterize the effect of polyhydramnios on maternal serum lithium levels and to evaluate the potential clinical implications for therapeutic monitoring and maternal and fetal safety.

**Methods:**

Women attended in a perinatal psychiatric outpatient clinic of a single tertiary university hospital and treated with lithium (in monotherapy or politherapy) in the perinatal period were evaluated for eligibility to participate in this retrospective observational cohort study (November 2006-December). HCB/2020/1305). Study included demographic, psychiatric, obstetric, and serum lithium concentrations data for each pregnancie obtained from the hospital medical records.Serum lithium concentrations were determined by means of an AVL 9180 electrolyte analyser based on the ion- selective electrode (ISE) measurement principle: Limit of quantification (LoQ) was 0.20 mEq/L and detection limit (LoD) was 0.10 mEq/L. Longitudinal pattern of serum lithium concentrations during perinatal period were studied using linear mixed models.

**Results:**

A total of 1,260 serum lithium concentration measurements were collected from 109 pregnancies in 95 women who received lithium treatment during the perinatal period. Polyhydramnios was observed in 11 pregnancies (10%) between gestational weeks 28 and 36, with two cases classified as severe (amniotic fluid index > 35 cm). One of these cases required amnioreduction.Screening for potential underlying etiologies was negative in all cases of polyhydramnios. Given a dose of 1000 mg, serum lithium concentrations decrease in women without polyhydramnios (n=98) on average 32.9 % in the first trimester of pregnancy, 32.2 % in the second, 21.0 % in the third and increase by 0.3 % in the first trimester of postpartum. On average, mothers with polyhydramnios (n=11) exhibit higher serum lithium levels compared to those without polyhydramnios, while the relative changes are similar in both groups. Based on the linear mixed model, controlling for period and lithium dose, the differences in serum lithium concentrations between pregnant women with (n=11) and without polyhydramnios (n=98) were not statistically significant (p=0.144).

**Conclusions:**

Our findings suggest that polyhydramnios does not affect serum lithium concentrations during pregnancy. Further prospective studies are warranted to develop individualized lithium monitoring protocols throughout the perinatal period.

**Disclosure of Interest:**

None Declared

